# Potential and limitations of socially assistive robots to support malnutrition care: a critical reflection by dietitians

**DOI:** 10.3389/fdgth.2026.1747543

**Published:** 2026-10-07

**Authors:** Janna W. Alberts, Martijn H. Vastenburg, Henk Herman Nap, Wijnand A. IJsselsteijn

**Affiliations:** 1 Human-Technology Interaction Group, Department of Industrial Engineering and Innovation Sciences, Eindhoven University of Technology, Eindhoven, Netherlands; 2 Vilans, National Centre of Expertise for Long-term Care, Utrecht, Netherlands; 3 Donders Centre for Cognition, Radboud University, Nijmegen, Netherlands

**Keywords:** care network, dietitians, malnutrition, older adults, research through design, SARS, contextmapping

## Abstract

Nutrition is essential for the physical and psychological well-being and health of older adults. Malnutrition negatively affects both their physical and mental health and their overall quality of life. Dietitians face challenges in supporting older adults due to time constraints, which can limit the continuity of care. While previous research highlights the potential of assistive technologies to help reduce malnutrition, such as socially assistive robots (SARs), these approaches lack a holistic perspective and insufficiently address collaborative processes within the broader care network. This study presents the findings from a Research-through-Design (RtD) study in which eight dietitians from four practices participated in context-mapping sessions involving personas, generative design probes, ranking exercises, and speculative design activities to critically reflect on the potential role of socially assistive robots in supporting collaborative malnutrition care. The results were analyzed using reflexive thematic analysis. These results highlight key challenges in nutritional care, including the social nature of eating, the need for personalized support, and challenges within the care network. These findings informed potential roles for SARs, including reinforcing dietary advice, supporting shared decision-making, facilitating collaboration, and encouraging shared eating moments. Crucially, the findings suggest that the value of SARs depends less on adding new functionalities than on carefully positioning their role within collaborative nutritional care. This includes preserving older adults’ autonomy, clearly allocating responsibilities between dietitians and technology, supporting meaningful engagement between consultations, and maintaining appropriate professional boundaries.

## 1 Introduction

Malnutrition affects 28% of older adults in hospital, residential, and community settings across Europe (1). Malnutrition often results from a combination of direct factors (such as forgetfulness, poor appetite, and meal preparation challenges) and indirect factors (including chronic disease, cognitive impairment, and oral pain) that affect nutritional status, as outlined in the Determinants of Malnutrition in Aged Persons (DoMAP) model (2). Malnutrition has a significant impact on the well-being of older adults, reducing their quality of life and increasing the risk of being hospitalized or institutionalized at an early stage (3–6). Hence, improving their nutritional status positively impacts older adults’ well-being and independence (7–9). Dietitians provide nutritional care through assessment, diagnosis, intervention, monitoring and evaluation (10–12).

Guiding older adults may be challenging due to systemic limitations and reliance on informal care and support networks (5, 6, 8, 13–16). Addressing malnutrition requires team effort, with clear communication and defined roles (17, 18). Responsibility for care is increasingly shifting toward older adults and informal caregivers, who act as knowledge brokers within their care networks (8, 19–21). However, effective collaboration is frequently hindered by high workloads, suboptimal communication, unclear responsibilities, and the low prioritization of nutrition (10, 14, 22). This leaves older adults vulnerable to exacerbations of malnutrition (15).

Technology has the potential to support older adults and their care networks; however, its optimal role in nutritional care remains insufficiently defined. Digital applications that provide functions such as monitoring, reminders, dietary advice, or teleconsultation have shown promise in addressing malnutrition (23–26). In addition to these software-based interventions, more tangible technological solutions, such as smart spoons, dining trays, and socially assistive robots, may improve nutritional intake (27–31). In particular, socially assistive robots (SARs) have demonstrated potential as mealtime companions by encouraging eating behavior and providing social companionship. Additionally, a social companion may provide support with daily structure or with loneliness among older adults (24, 32–35).

Compared to static applications, the engaging and interactive nature of socially assistive robots (SARs) may enhance their potential impact (36, 37). Features such as voice, facial features, and responsive interaction can foster experiences of social connectedness, which may support long-term engagement and reduce feelings of judgment among users (38, 39). Older adults acknowledge the potential of SARs but emphasize that their effectiveness as social companions depends on feeling understood by the SARs and perceiving it as genuinely helpful (40). However, these technologies often fail to address the collaborative process between formal and informal carers and clients, which is crucial in the care network (40). These technologies may not always adequately meet end users' needs, particularly when the perspectives of older adults and their care networks are insufficiently considered (41–43). Therefore, it is important to explore the role that socially assistive robots (SARs) could play within the complex collaborative ecosystem of malnutrition care. This study addresses this gap by moving beyond a focus on functionality, usability, or acceptance, and instead examines how SARs might be meaningfully positioned within the care network. In particular, it explores the perspectives of care professionals, such as dietitians, on the potential role of SARs in supporting care practices.

To explore the potential role and limitations of socially assistive robots (SARs) in supporting malnutrition care, this study adopted a Research-through-Design (RtD) approach (44–48). RtD served as an overarching research approach, enabling the exploration of complex care challenges through design-oriented inquiry and critical reflection rather than the evaluation of predefined technological solutions (47). Within this framework, context mapping was used as the primary generative research method to elicit participants' experiences, uncover tacit knowledge, and stimulate reflection on the possible roles of SARs within collaborative malnutrition care (45, 46).

Building on earlier work by Alberts et al. (49) this study addresses the following research question: “How do dietitians conceptualize and evaluate the integration of SARs within collaborative malnutrition care?” This study is exploratory and aims to inform a conceptual design for the use of socially assistive robots (SARs) in nutritional care, highlighting where their use may be meaningful, limited, or even unwelcome.

## 2 Methods

### 2.1 Study design

This study adopted a Research-through-Design (RtD) approach to explore how dietitians conceptualize the role of socially assistive robots (SARs) within collaborative malnutrition care. Rather than evaluating an existing technological solution or eliciting opinions through conventional interviews alone, RtD was used as a generative research approach to support critical reflection on possible future care practices and technologies. Within RtD, knowledge is generated through the design and use of artifacts that enable participants to articulate tacit experiences, challenge existing assumptions, and collectively imagine alternative futures.

To operationalize this approach, we employed context mapping, a participatory design methodology that combines generative design activities with structured reflection. Participants were invited to move gradually from describing their current care practices toward envisioning possible future roles for SARs. Rather than treating the design activities as independent evaluation tasks, each activity was intended to reveal how dietitians reason about professional responsibilities, collaboration within the care network, and the allocation of tasks between humans and technology. This design-oriented inquiry generated qualitative insights into both the opportunities and the boundaries of integrating SARs into collaborative malnutrition care.

Given the exploratory nature of the study, the objective was not to design or validate a particular robot, but to develop a richer understanding of how dietitians position socially assistive technologies within nutritional care and which responsibilities they consider appropriate—or inappropriate—for technological support.

### 2.2 Participants

Eight dietitians participated in the RtD sessions. The participants (aged 27 to 49 years) had between 1 and 20 years of experience as dietitians, providing variation in perspectives across different stages of professional experience. They were part of four dietetic practices, with two dietitians in each practice. These practices, located in urban areas of the Netherlands, consisted of two to four dietitians each. Participants were recruited through the first author's professional network and the HAN University of Applied Sciences in Arnhem and Nijmegen. Due to the exploratory and qualitative nature of the study, depth of insight was prioritized over sample size. Participants were selected based on their experience with malnutrition among older adults. The final sample included eight dietitians who provide nutritional support to community-dwelling older adults. Data collection continued until sufficient richness and diversity of dietitians' perspectives had been obtained to address the research question.

### 2.3 Rtd session design

A total of four dyadic sessions took place, with two dietitians from the same practice participating in each session. This format was chosen to stimulate discussion and reflection between dietitians. Throughout the sessions, the first author facilitated the discussion and ensured that each participant had an equal opportunity to elaborate on their viewpoints. The aim was not to seek consensus but to encourage discussion and reflection. The first author conducted the sessions in Dutch, each lasting approximately 60 min. They took place face-to-face in the treatment rooms of the participating dietetic practices. The dietitians were instructed to think aloud and explain each step, design, or word they used or wrote throughout the reflective sessions.

The RtD session was deliberately structured in five phases to reflect on current practice by creating a persona, followed by an exercise to reflect on previously experienced challenges, and a ranking-and-drawing exercise to explore the potential role of SARs and express expectations for future care scenarios (Figure 1). The sessions followed a gradual progression from reflection on current care practices toward speculation about future technological support. This sequencing intentionally delayed the introduction of technology so that participants first articulated existing care challenges before considering possible technological interventions. Additionally, to avoid constraining participants' thinking with a predefined notion of form or functionality, we deliberately omitted the term “socially assistive robot”. Instead, the concept was introduced as a “food companion” or “assistant” to encourage open exploration of the potential role of a digital entity supporting older adults and their care network.

**Figure 1:**
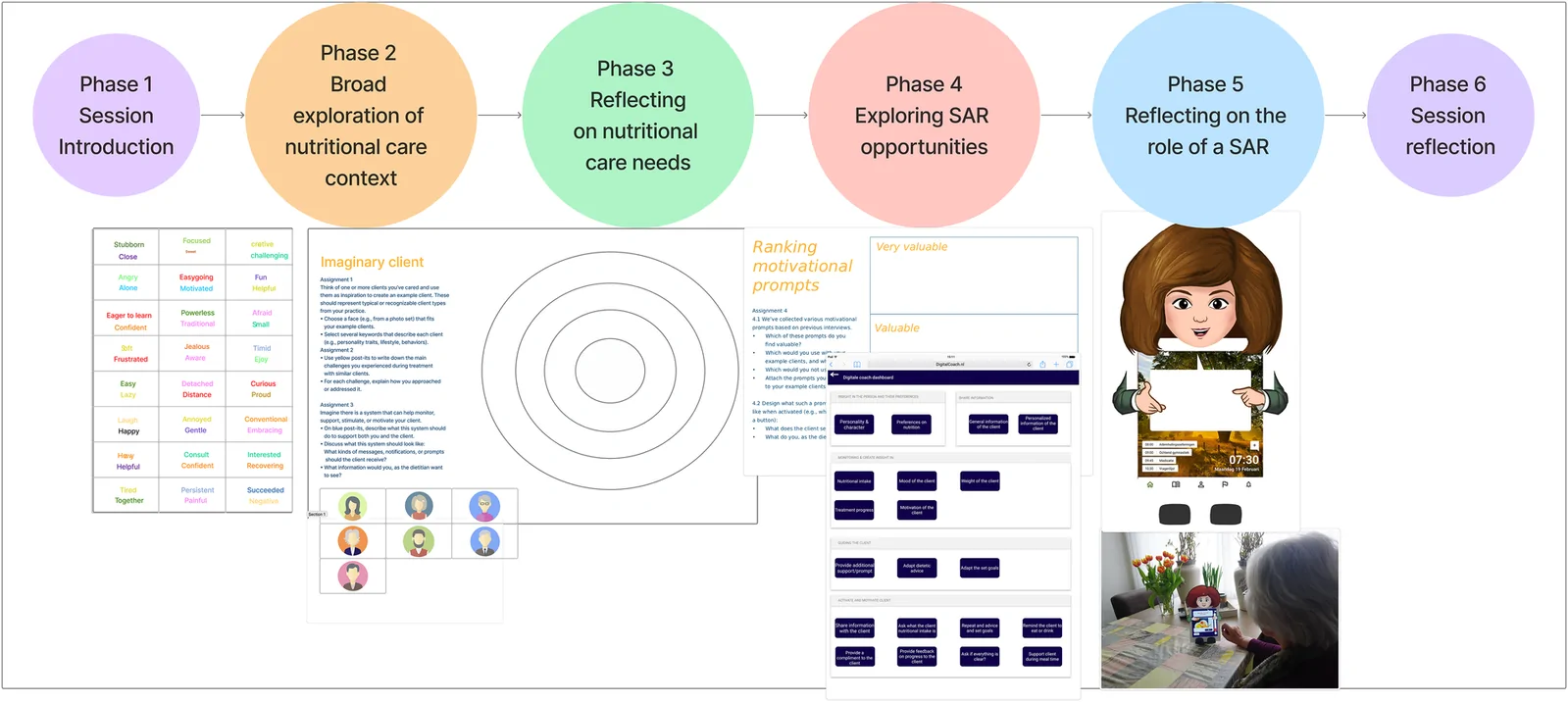
The RtD sessions were conducted in the dietetic practices. The sessions were divided in five phases, starting from the introduction to reflecting on the role of a SAR.

### 2.4 Design materials and probes

The RtD sessions employed a set of generative design materials that were developed to progressively support participants in reflecting on current nutritional care practices and envisioning the future role of socially assistive technologies. Rather than functioning as evaluation instruments, these materials acted as epistemic probes that encouraged participants to articulate tacit knowledge, challenge existing assumptions, and externalize their expectations regarding future care practices. Together, the materials guided participants from reflection on present-day experiences towards increasingly concrete conceptualizations of the role of SARs within collaborative malnutrition care.

#### 2.4.1 Persona toolkit

The session began with the creation of an imaginary older adult experiencing malnutrition (Figure 2). Participants constructed personas using a template, character stickers, and trigger-word cards describing personality traits, lifestyle characteristics, and behavioural tendencies. Rather than representing a specific client, the personas served as boundary objects that grounded subsequent discussions in realistic care situations while encouraging participants to draw upon their own professional experience.

**Figure 2:**
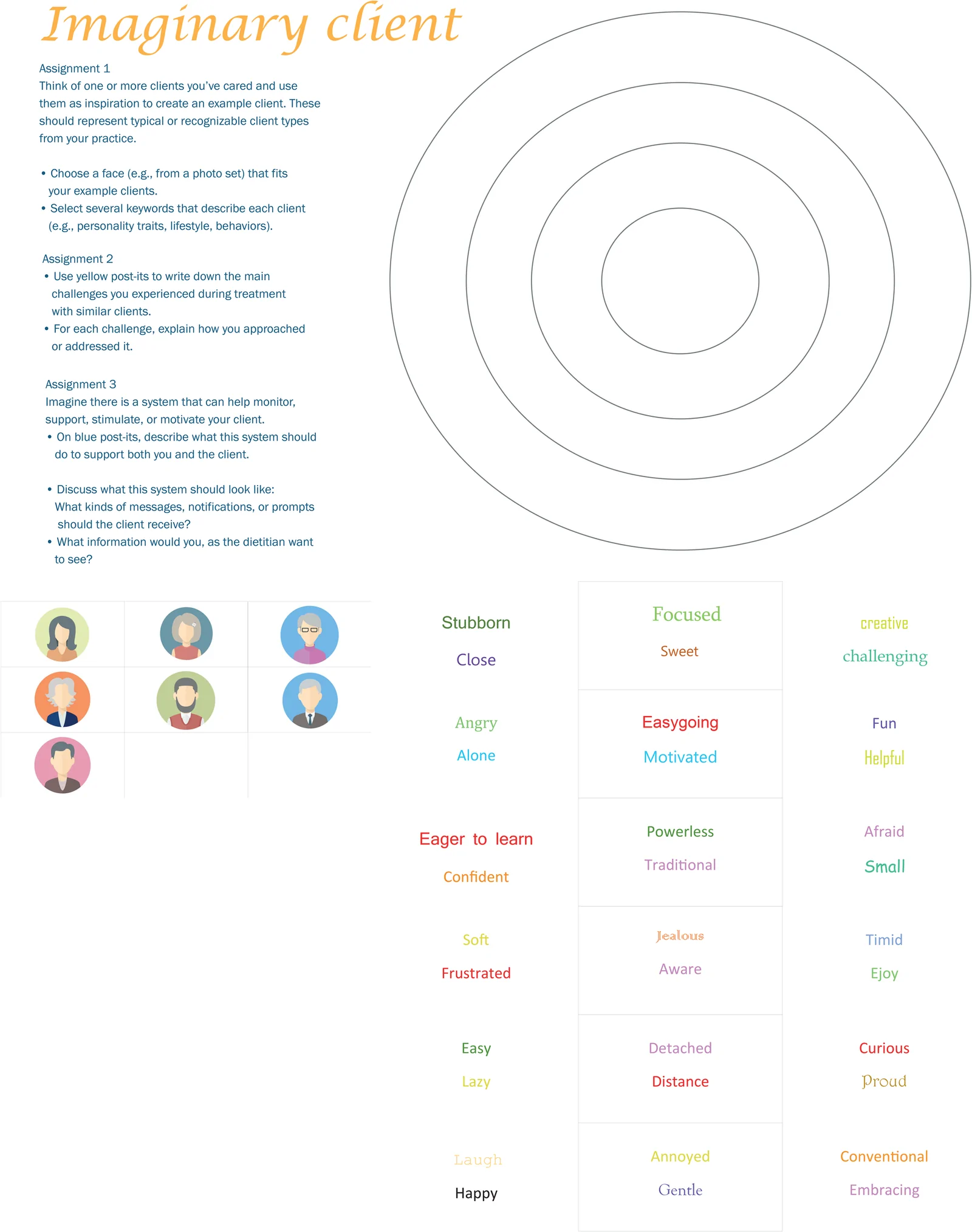
Persona template used by dietitians to describe an imaginary client with their own struggles and challenges based on their previous experiences, plus an example of the stickers used to create the persona.

Following persona creation, participants identified previous challenges encountered during nutritional care using Post-it notes. This activity elicited rich descriptions of everyday practice, revealing not only nutritional problems but also interpersonal, organizational, and contextual challenges that shape dietetic care.

#### 2.4.2 Intervention dashboard

An intervention dashboard (Figure 3) presented a range of possible technology-supported intervention buttons derived from previous qualitative work on assistive technologies for malnutrition care (49). The themes, subthemes, and underlying qualitative findings from this study were translated into abstract technology-supported intervention opportunities, which were subsequently formulated as concise intervention buttons. Rather than representing a validated set of robot functions, these intervention buttons were intentionally formulated as provocative design concepts.

**Figure 3:**
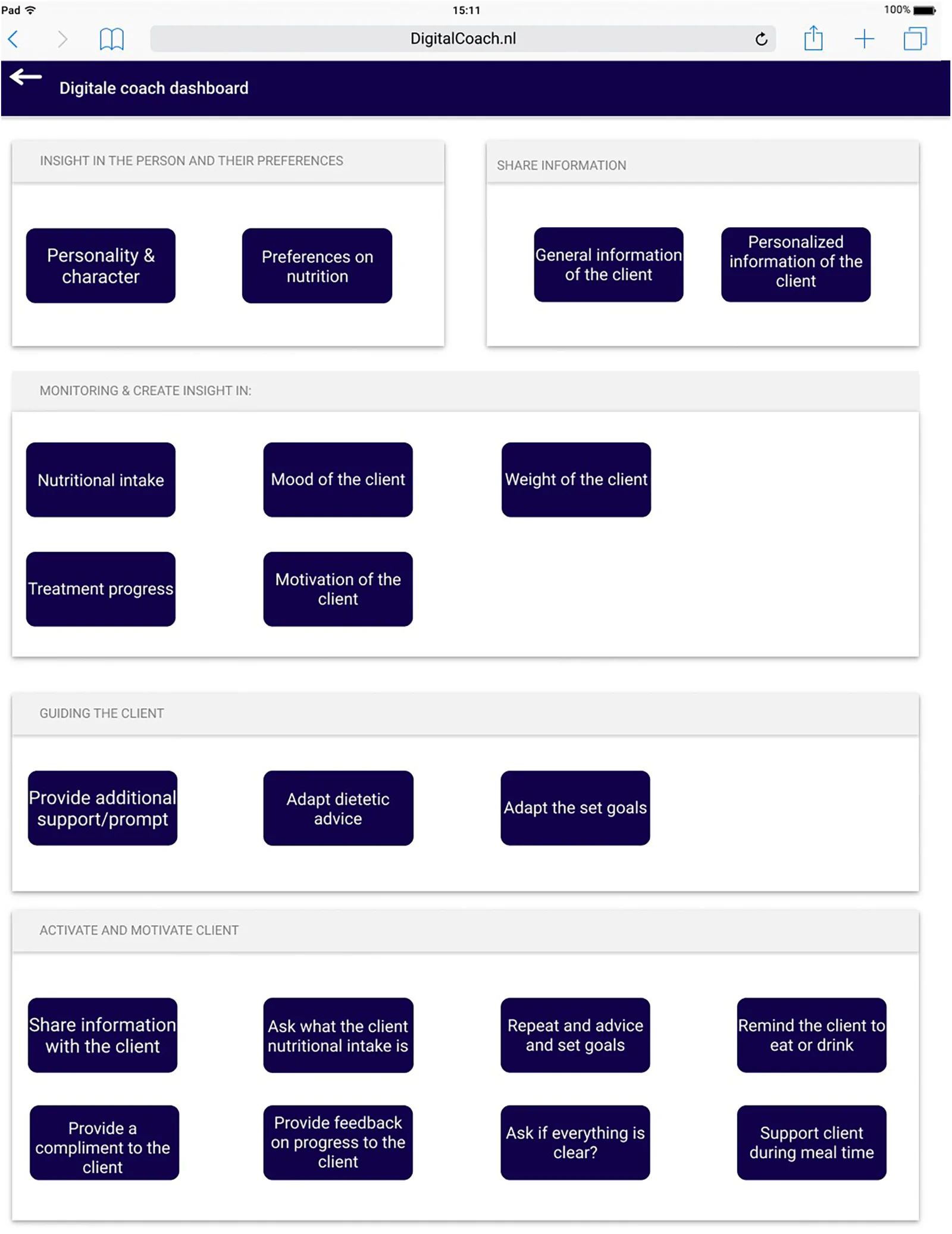
An intervention dashboard presenting potential intervention buttons that may or may not support the dietetic journey. These buttons are based on previous insights by Alberts et al. (44).

Participants were invited to discuss, prioritize, modify, reject, or expand these concepts. This encouraged reflection not only on which interventions might be valuable, but also on why certain responsibilities were considered appropriate—or inappropriate—for technological support. The dashboard therefore functioned as a mechanism for exploring participants' implicit assumptions regarding the allocation of tasks between dietitians, older adults, informal caregivers, and technology.

#### 2.4.3 Prioritization board

Participants subsequently organized the intervention concepts into categories representing high, moderate, or low value (Figure 4). Rather than generating quantitative rankings, this activity was designed to stimulate discussion and negotiation between participants.

**Figure 4:**
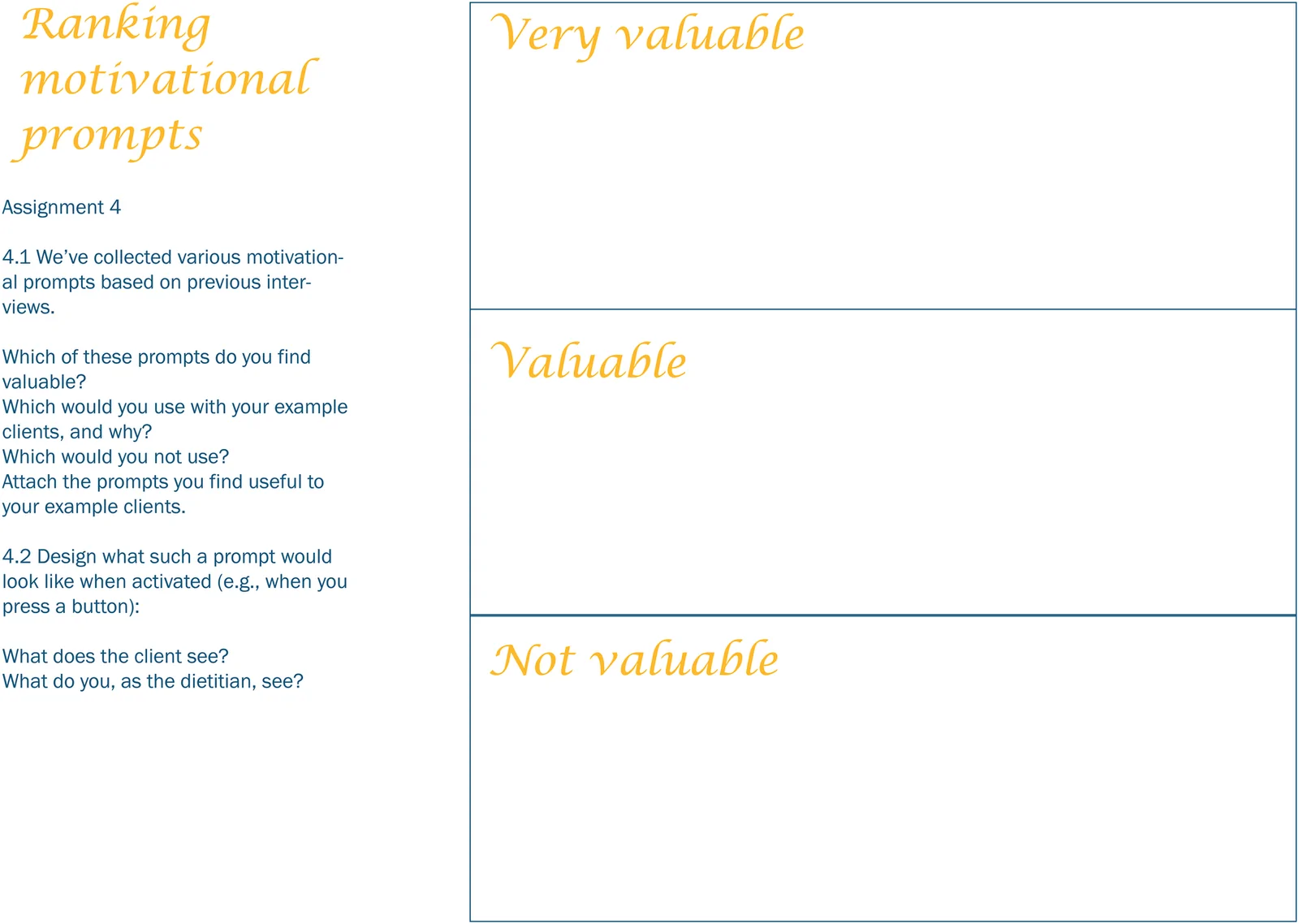
Ranking motivational prompts based on the importance of how the intervention buttons previously shown in Figure 3 should be ranked when applied in their own context.

The conversations surrounding prioritization proved more informative than the resulting rankings themselves, revealing how participants weighed clinical value, feasibility, ethical considerations, and professional responsibilities when considering technological support.

#### 2.4.4 Lizz: speculative SAR probe

A speculative prototype named *Lizz* was introduced to support reflection on the possible role of an embodied socially assistive technology within nutritional care (Figure 5). In addition, other SARs currently available on the Dutch market were introduced, including Paro, Tessa, and Sara (50–52). Lizz was not presented as a finished product or as a prototype to be evaluated, but as an intentionally low-fidelity design probe representing one possible future embodiment of a SAR. Although intentionally under-specified, its embodied form and conversational interaction provided sufficient social presence to stimulate reflection on the potential social and supportive role of a SAR, rather than on specific technological capabilities.

**Figure 5:**
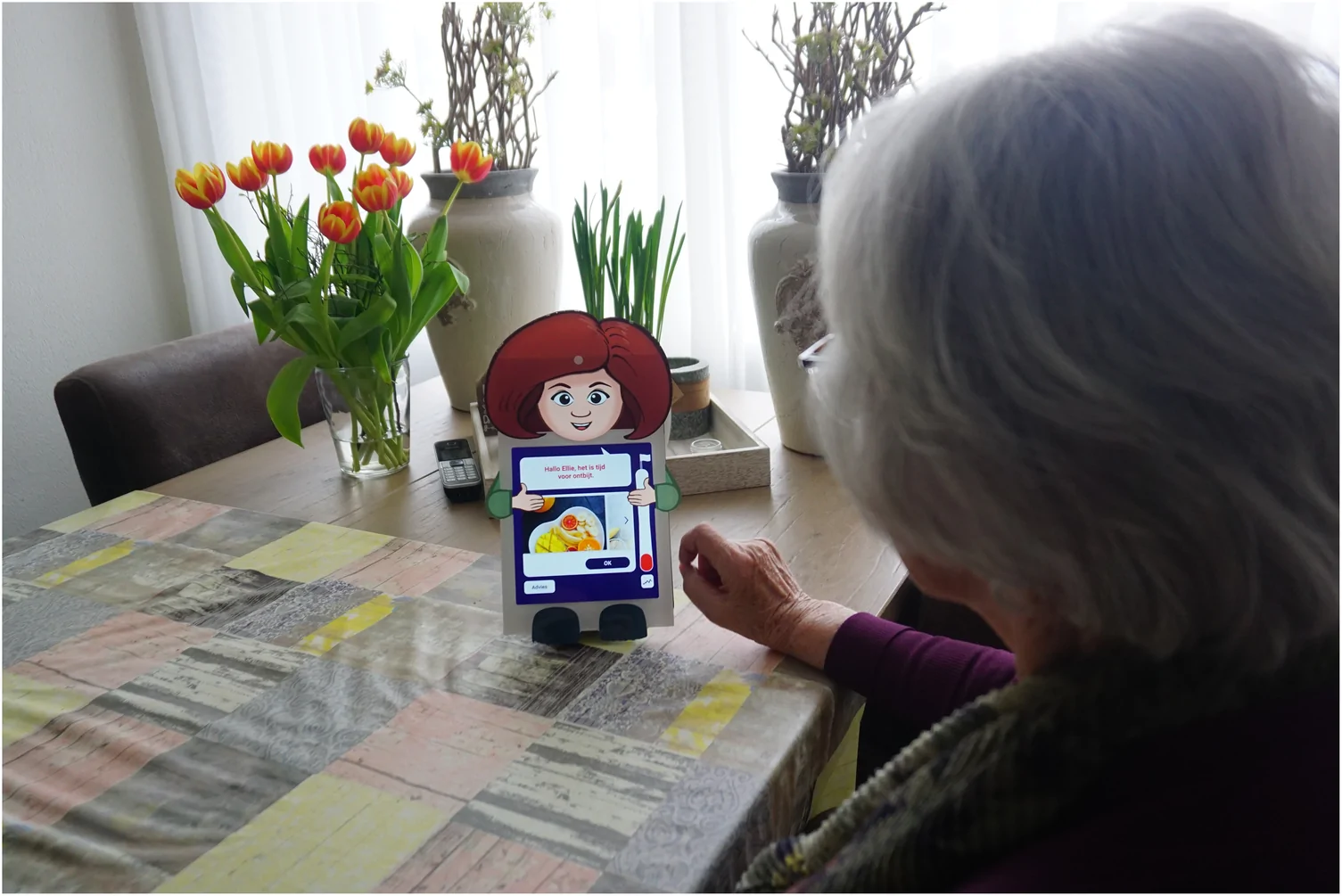
The socially assistive robot prototype, lizz, used as a probe in the RtD-sessionas an example of potential SARs.

Several design choices were made deliberately. Functional capabilities were intentionally left under-specified to avoid anchoring participants to particular technological solutions and instead encourage reflection on desired roles and responsibilities. Likewise, Lizz adopted a stylized, cartoon-like appearance rather than a highly realistic embodiment, providing sufficient social presence to facilitate discussion while minimizing attention to aesthetic preferences or technological realism.

Importantly, Lizz was introduced only after participants had reflected extensively on current care practices and collaboratively identified challenges within nutritional care. This sequencing was intended to ensure that subsequent discussions remained grounded in existing care practices rather than being driven by the technological characteristics of the probe itself.

As with any design probe, Lizz inevitably shaped participants' reflections. Rather than viewing this as a source of bias, we consider this framing an inherent characteristic of generative design research, where deliberately designed artifacts function as catalysts for articulating otherwise tacit assumptions regarding future technologies.

#### 2.4.5 Concept sketch template

Finally, participants used a design template (Figure 6) to externalize their own conceptualizations of future SARs. Building upon the preceding discussions, they were invited to sketch interactions, formulate prompts, describe desired behaviours, and indicate information flows between the older adult, the care network, and the dietitian.

**Figure 6:**
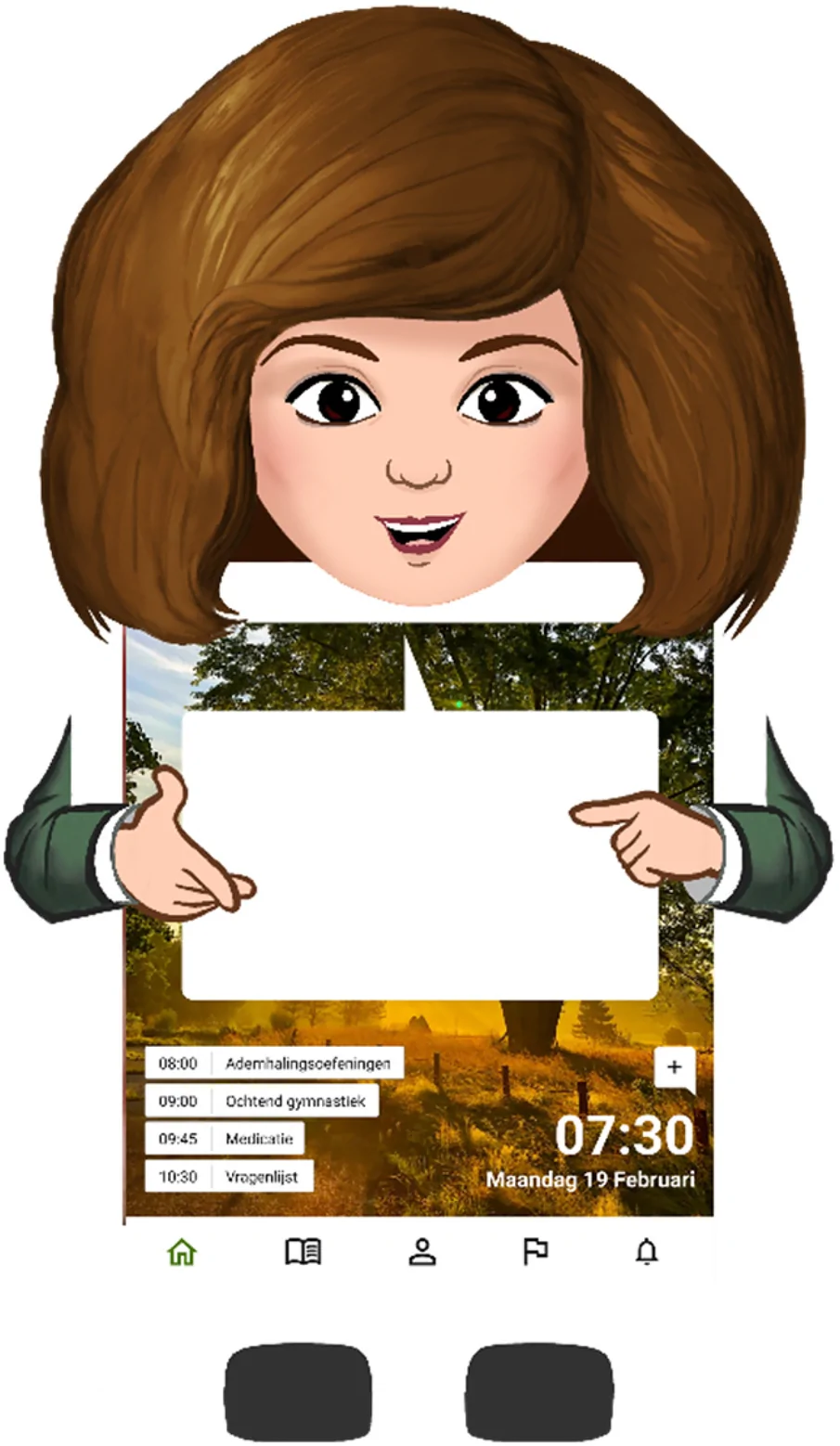
Concept sketch template used to explore participants ‘envisioned roles and interaction for SAR.

The purpose of this activity was not to generate design specifications for future robots, but to externalize participants' underlying mental models regarding the appropriate role, responsibilities, and boundaries of socially assistive technologies within collaborative nutritional care.

### 2.5 Data collection

Data were collected during four face-to-face dyadic RtD sessions. Each session was audio-recorded and transcribed verbatim. During the sessions, participants engaged in different generative design activities, including creating a persona, ranking intervention buttons, and conceptualizing the role of the SAR probe Lizz. All completed design materials were collected alongside the interview transcripts and used to support interpretation during the reflexive thematic analysis.

### 2.6 Data analysis

All RtD sessions were analyzed using reflexive thematic analysis (53, 54). A latent, inductive approach was applied to critically examine the role of SARs. The analysis drew on both the interview transcripts and the artefacts generated during the RtD sessions, including personas, intervention rankings and design sketches (44–48).

The themes were developed through several steps. The data was organized using MAXQDA 2022 (55). After coding, we clustered related codes into subthemes that captured recurring patterns in the data. The personas, rankings, sketches, and other design outputs were subsequently examined alongside these subthemes to enrich their interpretation and identify converging patterns across the different activities. This helped ensure that nuances expressed through the different activities were reflected in the developing themes. We then examined how these subthemes connected at a broader level and organized them into four overarching themes. The research question and the aim of the exploration influenced the themes created. The main themes reflect broader challenges and aspects within the nutritional care process, while the subthemes are more concrete experiences and expectations as expressed by the participating dietitians.

### 2.7 Positionality

This methodology was chosen because it supports interpretive, iterative engagement with qualitative data, which aligns with the exploratory nature of this study. Rather than focusing on coding consensus, this approach recognizes the researcher's interpretive role and supports analysis of both explicit statements and implicit assumptions about SARs. To ensure transparency, we explicitly acknowledge our interpretive standpoint. We approached the analysis from a contextualist perspective, recognizing that dietitians' experiences are shaped by their daily practice within the healthcare system. The first author's background in human-centered design, gerontechnology, and aging shaped the interpretive lens, particularly in understanding the dynamics of the care network (56). The aim of the analysis was not to reach full consensus but to engage in reflexive dialogue about the emerging themes and to critically reflect on how interpretations were formed.

## 3 Results

Four main themes and thirteen subthemes were identified, as shown in Figure 7, which offers an initial direction for the role SARs can play in supporting the malnutrition care process, benefiting both older adults and their care networks. The figure displays the themes and sub-themes, color-coded for clarity.

**Figure 7:**
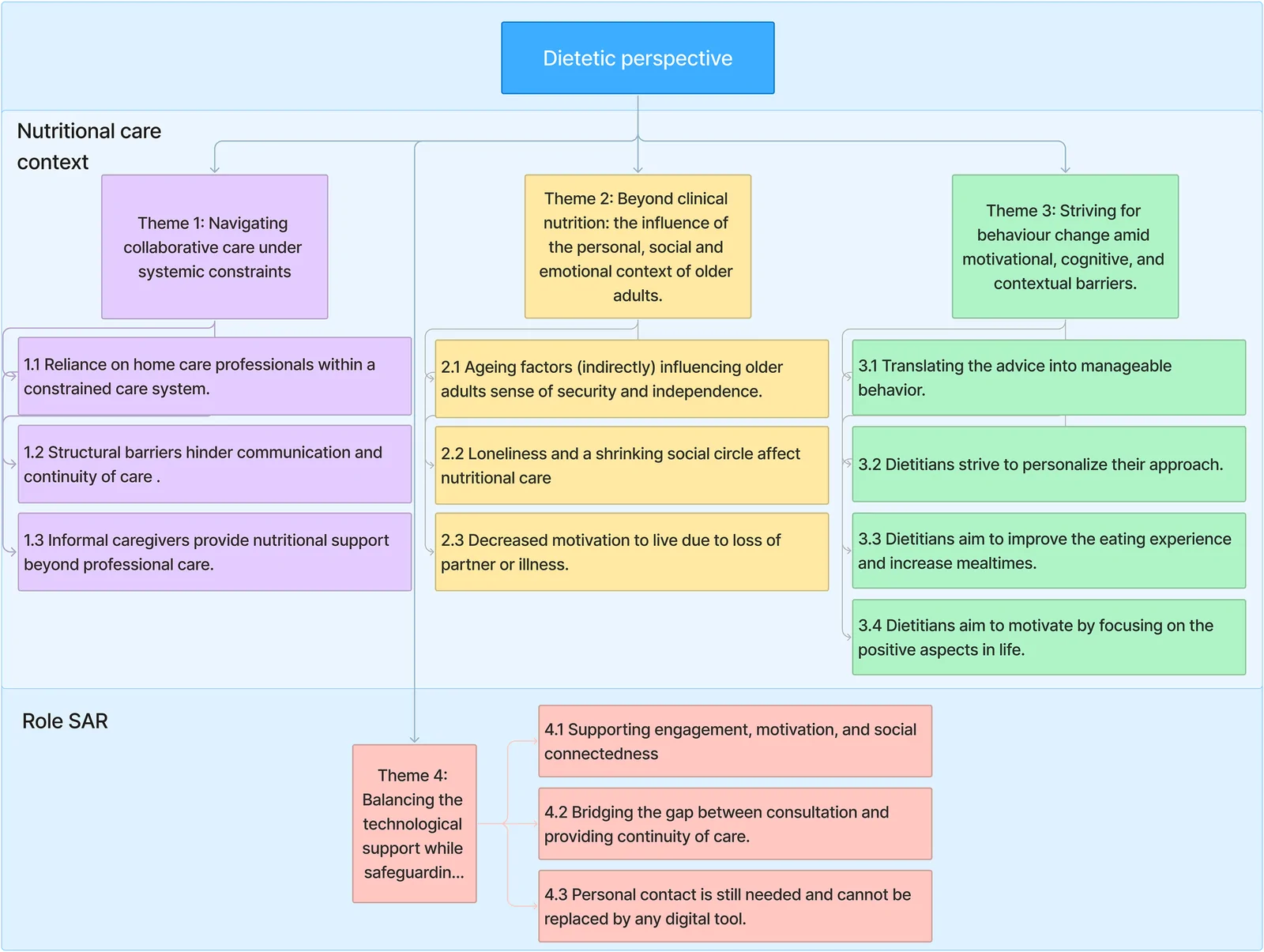
The thematic analysis supports defining the opportunities and challenges towards the role SARs can provide in the care of malnutrition, supporting the older adult and the collaborative process within the care network.

Alongside the thematic analysis, brief reflections from the probes have been integrated to contextualize the findings.

### 3.1 Reflections from the probes

The personas revealed recurring patterns across five categories. Dietitians emphasized older adults' social vulnerability, including loneliness, limited family involvement, and reliance on home care professionals, who may at times constitute their primary social contact. Nutritional vulnerability was also highlighted, including dependence on oral nutritional supplements and meal services. Older adults were frequently described as forgetting to eat due to reduced appetite or altered taste, often associated with cognitive decline. In addition, physical decline, manifesting as pain, fatigue, and reduced mobility, was seen to contribute to feelings of powerlessness, particularly when cognitive functioning remains relatively intact. The personas further reflected a contrast between highly motivated individuals and more passive older adults who have lost motivation. Finally, older adults were frequently characterized as habit-driven and often resistant to changes in nutritional routines.

Building on the insights generated during the persona activity, participants subsequently reflected on how a SAR might support these identified challenges. Dietitians envisioned a support system primarily functioning as a reminder, motivational coach, monitoring tool, and mealtime companion. Key functionalities included prompting eating and drinking, reinforcing dietary advice, and providing insight into nutritional intake, particularly regarding oral nutritional supplements that are frequently overlooked. Additional roles included practical support in meal preparation and fostering companionship during meals to reduce loneliness and enhance the meaning of eating moments.

The intervention dashboard further refined these reflections by asking participants to prioritize different technology-supported intervention opportunities. The prioritization exercise reinforced these insights. Dietitians consistently ranked reminding, reinforcing advice, motivating behavior, and monitoring nutritional intake and weight as the most valuable functions. Positive reinforcement, such as compliments and supportive prompts, was also highly valued, whereas autonomous goal-setting or dietary advice was consistently ranked as less important, reflecting participants' view that responsibility for nutritional guidance should remain with the dietitian. This distinction was subsequently reflected in the concept sketches and envisioned SAR interactions, which focused on positive reminders, simple check-ins on intake and well-being, and encouraging feedback, often supported by visual or auditory cues to facilitate interaction.

### 3.2 Thematic analysis

The RtD sessions generated insights into both the context of collaborative malnutrition care and dietitians' reflections on the potential role of SARs. Rather than representing separate findings, the four themes build upon one another. Themes 1–3 describe the collaborative, personal, and behavioural challenges that shape everyday nutritional care. These findings, which emerged from the first two phases of the RtD sessions, provide the context for interpreting the design opportunities and boundaries of SARs. Building on this foundation, Theme 4 presents the findings from the design-oriented phase of the RtD session, following the introduction of the intervention dashboard and the SAR probe. During this phase, dietitians were invited to critically reflect on the potential role of SARs within the nutritional care process, gradually moving from understanding the care context towards exploring design opportunities.

### 3.3 Theme 1. Navigating collaborative care under systemic constraints

Participating dietitians emphasized their reliance on the broader care network and noted several challenges within the healthcare system that limit the support they receive from the care network, including time constraints and restrictions on the type of care tasks they can perform. Dietitians also underscored the critical role of informal caregivers in supporting nutritional care.

#### 3.3.1 Reliance on home care professionals within a constrained care system

Dietitians emphasized that, although they are the primary experts in nutritional care, effective support depends on close collaboration with the broader care network. In particular, they rely on home care professionals to monitor older adults, recognize signs of nutritional decline, and reinforce dietary advice in everyday practice. However, participants observed that this supportive role has become increasingly difficult to fulfil due to workforce shortages, limited capacity, and reimbursement constraints. They stressed that the challenge is not primarily the willingness of home care professionals to support nutritional care, but the more systemic constraints such as reimbursement limitations and workforce shortages. This growing imbalance was a source of frustration for the participating dietitians, who described situations in which frail older adults were left unsupported due to both limited professional care and the absence of informal caregivers, as described in Session 1:.

Session 1, Dietitian 1 & 2: D1: “Yes, there are these ladies that are still living at home, though they are so undernourished that if you would slam the door too hard, they would fly off the couch. D2: Yes indeed, it is so sad. D1: and that makes it frustrating.”

Participants described a healthcare system that, in their view, is failing to adequately support older adults, especially when informal caregivers are absent. One particularly striking example highlighted the serious consequences of this lack of support:

Session 2, Dietitian 1: “She was a single woman with dementia and … she forgot, she just passed away … she forgot to eat due to her dementia, and there was nobody in her network that could remind her to eat, and the limited home care, and a small care network no friend, no family …”

#### 3.3.2 Structural barriers hinder communication and continuity of care

Effective collaboration with home care professionals was frequently hindered by structural and organizational barriers. Participants described fragmented communication caused by incompatible electronic health record systems, rotating care teams, uncertainty about who to contact, and the high threshold for interprofessional communication. As a result, dietitians were often uncertain whether nutritional advice reached the professionals providing day-to-day care or was consistently reinforced in practice. As one participant explained in session 2:

Session 2, Dietitian 2: “There are a lot of rotations due to holidays … so agreeing on a specific plan with a home care professional is challenging, even though they put it in their treatment plan.”

Participating dietitians expressed the hope that a SAR could help address some of these described gaps. Not only activating the older adults with reminders but also prompting the home care professionals to reinforce the dietary advice given.

#### 3.3.3 Informal caregivers provide nutritional support beyond professional care

Dietitians emphasize that informal caregivers are crucial in supporting older adults with malnutrition. As one dietitian explains, their absence shows how critical informal caregivers are; without them, older adults may not have the proper nutrients in their fridge to improve their nutritional intake. This becomes even more crucial when the older adult is suffering from cognitive decline, such as Alzheimer's disease:

Session 2, Dietitian 1: “Someone with cognitive problems, you cannot really agree on things, someone with an informal caregiver can at least make an appointment and arrange appointments with the hospital … also the social aspect… they can check how someone is doing …. The informal caregivers manage quite a lot.”

However, even willing informal caregivers may feel insecure or uncertain about how to apply the given advice effectively.

Collectively, in terms of potential design relevance, the insights described under Theme 1 suggest that dietitians do not primarily seek technologies that replace professional care, but rather technologies that compensate for discontinuities within the care network. The described fragmentation, workforce shortages, and communication barriers position SARs not as autonomous care providers, but as supporting actors that can reinforce continuity between consultations, facilitate communication within the care network, and help ensure that nutritional advice remains visible and actionable in everyday care.

### 3.4 Theme 2: beyond clinical nutrition: the influence of the personal, social and emotional context of older adults

#### 3.4.1 Ageing factors (indirectly) influencing older adults' sense of security and independence

The dietitians described the aging process as significantly influencing the quality of life of older adults. The (chronic) illnesses, decreased physical abilities, lack of energy, and (constant) physical pain negatively affect their daily activities, thereby affecting their nutritional intake. According to the participants, clients may feel overwhelmed by these factors and experience a loss of independence and control over their lives. The client feels frustrated that their bodies are “abandoning” them while their minds are still healthy, as explained:

Session 1 Dietitian 2: “Often powerlessness, …[..]. that their body is giving up while their mind is still sound, I see that a lot, …, it's like a chain reaction of events … getting older, not able to move, their bones…. and it is all overwhelming…. Getting into a negative spiral.”

#### 3.4.2 Loneliness and a shrinking social circle affect nutritional care

The physical decline leading to limitations in daily activities, such as grocery shopping or visiting a friend, can significantly affect older adults' social contact. When they are not able to drive or go shopping, their social world shrinks as highlighted:

Session 2, Dietitian 1: “Their world gets smaller, their movements are limited, they don’t leave the house, they cannot drive anymore, they don't have coffee with others.”

As described by the participating dietitians, eating alone further adds to the older adults' disinterest in meals. Many stop making an effort to cook or set the table when living alone. These results were also reflected in the created personas, in which dietitians highlighted aspects such as loneliness as part of their clients' personas as well as the need for interaction and personal contact.

#### 3.4.3 Decreased motivation to live due to loss of a partner or illness

The loss or illness of a partner significantly affects their daily interactions, emotional well-being, and instrumental support. This can have such a profound emotional impact that people may lose their motivation to live:

Session 3 Dietitian 1: “Such a story in which there is no cure for his wife had such an impact on him. While he also has Parkinson's disease, you notice his will to live just disappears. ”

Dietitians experience a big contrast among their clients: one is highly motivated, doing everything in their power to increase their dietary intake; others may be passive in their care process and wait for the dietitians to define what to do.

Session 2, Dietitian 1: “Yes, this lady is passive. If I tell her she has lost a lot of weight, what do you think? (Their response) Yes, I do not mind it. I cannot influence it … She did not care…”

Dietitians try to respond with positivity, encouragement, and small wins. They aim to spark new habits, despite resistance and deep-seated beliefs. This was also reflected in the probe activities, where dietitians emphasized motivation as a key facilitator when constructing personas and designing SAR prompts.

In terms of design implications, the insights found under this theme suggest that nutritional care extends well beyond monitoring dietary intake alone. Because eating is embedded within older adults' social relationships, emotional well-being, and everyday routines, dietitians envisioned future SARs as technologies that should acknowledge these broader dimensions of care. Rather than solely monitoring nutritional status, participants described opportunities for SARs to encourage shared eating experiences, provide motivational support, and reinforce meaningful daily routines.

### 3.5 Theme 3: striving for behaviour change amid motivational, cognitive, and contextual barriers

#### 3.5.1 Translating the advice into manageable behavior

Beyond defining appropriate nutritional goals, dietitians described the difficulty of translating advice into concrete actions that older adults can understand, remember, and apply. However, participating dietitians encounter barriers, clients struggle to understand and recall the advice, lack the cognitive ability to act, or don't have the right nutritional products at home:

Session 1, Dietitian 2: “and it is all doable, it is executable, of course you give advice, but it should be doable for the client, such as grocery shopping and other things …”

Dietitians often struggle to balance the need to give thorough guidance with the risk of overwhelming the client. They simplify explanations, emphasize the personal benefits of change, and hope to encourage the older adult to follow. Participants search for explanations, such as increased energy or strength, to make the advice more compelling. Repeating the reason why they are changing their behavior and explaining the significance of the change will help to better understand what they are doing it for.

Session 2, Dietitian 1: “You always try to explain … it's not wrong what you are doing… but if you do this, then you will gain more energy, more proteins, you will gain strength… it's about explaining what effects it (the advised diet) can have on the problems they encounter.”

Dietitians not only provide nutritional guidance but also act as facilitators, offering strategies to clarify the advice and make the required behavior change realistic and achievable.

#### 3.5.2 Dietitians strive to personalize their approach

In addition to supporting the implementation of the advice, dietitians emphasized the importance of tailoring nutritional goals to the individual's nutritional status, preferences, needs, and context. One-size-fits-all advice will not lead to the necessary behavior change. The participants strive to align guidance with personal preferences and dietary needs. Dietitians acknowledge that standardized information available on nutritional platforms or websites often does not fit specific situations:

Session 2 Dietitian 1: “General information is not useful. Two portions of fruit per day, that is general advice, but if someone manages to eat one, it is not the most important; more important is that they gain weight and eat enough protein, than I would take one portion of fruit for granted.”

The participants emphasized the importance of a person's quality of life, especially when clients are nearing the end of their lives. In some cases, supporting comfort and personal values may take precedence over nutritional improvements:

Session 4: Dietitian 2: “Yes, that is sometimes difficult to define. How much do you still want to intervene?” Dietitian 1: “For us, it is also a challenge; the quality of someone's life should be the most important; you may project everything on a person; sometimes, you may need to accept less.”

#### 3.5.3 Dietitians aim to improve the eating experience and increase mealtimes

Decreased appetite and taste buds influence older adults' willingness and eagerness to eat. Participating dietitians focus on making meals more enjoyable by enhancing flavors, providing appealing recipes, or suggesting meal services. Participants sought to increase meal moments throughout the day, for example, by adding snack times. This challenge was also reflected in the probe activities, where dietitians emphasized the difficulty of increasing meal moments and explored ways to support more frequent eating throughout the day. However, memory cognitive problems prevent the older adult from remembering to eat:

Session 1, Dietitian 2: “When they finally receive a meal service, and they finally enjoy a meal with taste, I can help them with it, and I can finally support them.”

#### 3.5.4 Dietitians aim to motivate by focusing on the positive aspects of life

Dietitians emphasized the importance of motivating older adults through self-reflection, positivity, and/or environmental changes such as eating together. Supporting self-reflection may be a first step for the older adult to realize the importance of their nutrition. Involving family members, peers, or healthcare professionals during meal moments can also add a social value, leading to a more supportive and enjoyable atmosphere, as explained:

Session 1, Dietitian 1: “It's about motivating each other, as long as I can keep on motivating the client during the consults, that they really see the progress by, for example, measuring their grip strength, … it's all about the nice things, the improvements I try to focus on next to the gain weight.”

Together, the findings under this Theme indicate that the greatest opportunity for SARs does not lie in generating dietary recommendations themselves, but in supporting the difficult process of translating professional advice into sustainable everyday behaviour. Dietitians therefore conceptualized SARs primarily as behavioural reinforcement technologies, capable of repeating advice, encouraging adherence, monitoring progress, and supporting motivation between consultations while leaving interpretation and adaptation of treatment to the dietitian.

### 3.6 Theme 4: balancing the technological support while safeguarding the human role in the nutritional care process

#### 3.6.1 Supporting engagement, motivation, and social connectedness

Supporting engagement and motivation during the nutritional process. Dietitians described loneliness as negatively affecting nutritional intake. Although they encourage shared meals with family members or peers, they recognized this is not always feasible. Participants emphasized that eating with others can increase motivation to eat and make mealtimes more enjoyable, highlighting the importance of the social experience of eating alongside nutritional support. In situations where shared meals were not possible, some participants suggested that a SAR could offer companionship if it were able to engage in meaningful, responsive interactions. The tool should “listen” to the person, have a dialogue, and respond with real answers, not predefined statements.

Session 4 Dietitian 2: “Yes, just a little company, giving attention, less lonely, yes, if someone is alone, you just want someone to be there … you often see that people get lonely when they are alone…in company, you notice that people start eating better… I assume that the robot cannot join to eat a sandwich?”

Dietitians envisioned SARs as reinforcing nutritional behaviour through positive encouragement, compliments, reminders of goals and mealtimes, and repetition of previously discussed advice. Across the probe activities, prompts were consistently framed in a supportive and encouraging manner, aiming to reinforce dietary advice between consultations and facilitate communication with the dietitian.

Session 2, Dietitian 2: “Motivating, repeating the advice and goals, it (the socially assistive robot) might motivate them to follow the advice if it tells you, it is important to take your nutritional drinks since it will help you regain your strength. Then you might be able to go back to the billiards evenings.”

Participants emphasized acknowledging small improvements to sustain motivation, recognizing that nutritional care is not only about weight gain but also about the older adult's experience of eating. *Session 3, Dietitian 1: “I think mainly motivating the client by giving compliments that can indeed be valuable, it encourages people to eat more.”*

In addition, dietitians considered repetition an important strategy for reinforcing advice between consultations. Participants envisioned SARs providing reminders about goals, mealtimes, and nutritional advice, particularly for older adults experiencing cognitive decline or reduced appetite.

Session 2, Dietitian 1: “help people remember, because then they are busy with something, even if it is reading a book … it is just forgotten very quickly and before they know it, it is already …. Reminding them is the most important thing.”

#### 3.6.2 Bridging the gap between consultation and providing continuity of care

With only three reimbursed hours per client per year, participating dietitians feel constrained. A SAR could bridge the gap between consultations by monitoring a person's diet, weight, weight changes, and emotional and mental well-being, enabling timely and tailored interventions. Additionally, changes in the person's circumstances also affect nutritional intake and are essential to monitor. In selecting the buttons, all dietitians chose a button focused on monitoring nutritional intake or tracking weight changes in older adults to better anticipate necessary adjustments.

Session 2, Dietitian 1: “Imagine someone has this tool (SARs) and it asks (the older adult) to fill in their mood for that day, an emoticon, and imagine you get and message that someone has been down the whole day and therefore hasn't been eating, you might be able to actively respond to it, like asking why have you been down, or maybe also why have you been so happy.”

Additionally, dietitians reported that many older adults hesitate to reach out and ask for help, fearing they will be a burden. This barrier prevents older adults from signaling when they need additional help.

Session 3, Dietitian 1: “How does it influence her? Well, she is insecure and doesn't dare to ask for help; in her case, this means she doesn't have the right nutritional products in her home, and therefore she cannot really gain any weight.”

In an ideal world, dietitians would like more time and more frequent contact with clients to get to know them and tailor advice to their preferences and needs. However, as this perfect world does not exist, the next best thing is a digital tool to help increase contact with the client. The participating dietitians envision that a SAR could help normalize help-seeking behavior. Dietitians hope that older adults will be encouraged to communicate sooner when problems arise or if they have questions about the advice discussed. One of the dietitians designed a prompt in the SAR template asking the client whether they were able to apply the provided advice.

Session 2, Dietitian 2: “They do not want to be a burden, but if such a thing (a SAR) would suggest asking for help, maybe they might not feel a burden and know it is permitted to ask for help or to call…”

#### 3.6.3 Personal contact is still needed and cannot be replaced by any digital tool

The previous themes highlighted the importance of tailoring nutritional advice to the individual and building a trusting relationship to understand an older adult's preferences, needs, and personal circumstances. These considerations also shaped dietitians' views on the role of a SAR. Participants consistently emphasized that personal contact is an irreplaceable part of nutritional care. Face-to-face consultations were considered essential for understanding a person's context, experiences, and challenges, enabling dietitians to personalize advice and evaluate progress. Rather than replacing these interactions, dietitians envisioned SARs as supporting continuity between consultations by reinforcing advice, providing insight into changes in a person's situation, and facilitating timely communication when additional support is needed. At the same time, participants stressed that dietitians should remain in control of nutritional assessment, treatment decisions, and adapting advice to the individual. They also highlighted that the value and feasibility of SARs would depend on the older adult's personal preferences and digital skills, recognizing that such technology would not be suitable for everyone.

Session 2, Dietitian 2: “Then you also get to adjusting the goals. That is basically adjusting the nutritional advice. That is something we discuss at that moment, with input from the patient themselves. So that is not valuable (referring to the companion's function). For me, adjusting the goals and adapting the nutritional advice are the least relevant functions. Those are things we do together with the patient during the consultations.”

## 4 Discussion

Malnutrition adversely affects the health and well-being of older adults. Dietitians play a crucial role in tackling these issues but often face limitations, resulting in reliance on the care network's support, where collaboration is frequently sub optimal. Consequently, guiding older adults to improve their dietary intake remains difficult due to time constraints, interconnected factors, and systemic limitations within the healthcare system (10, 11, 16, 22).

This study employed a research-through-design (RtD) approach to explore the potential role of socially assistive robots (SARs) in malnutrition care. Through context mapping, personas, and design probes, dietitians explored their perspective by reflecting on their current experiences and challenges, and provided critical reflection on the role SARs may play. Rather than designing the perfect SAR, the aim was to explore how SARs can support the collaborative care process and where their role may be meaningful, limited, or even unwanted.

The findings reveal key tensions: (1) the fragmented responsibilities within the care network, (2) the balance between supporting and directing behavior change without undermining the autonomy of older adults, and (3) the irreplaceable value of human presence during socially meaningful moments, including at mealtimes. These tensions show that integrating SARs into malnutrition care is not only about providing the right functions within the SAR but also about exploring how SARs can be positioned within the relationships among dietitians, older adults, and their care networks. Taken together, these findings suggest that the role of SARs may extend beyond providing isolated functionalities. From a design perspective, SARs may be conceptualized as relational infrastructures that support interactions between older adults, dietitians, and the broader care network. Accordingly, we argue that this shifts the design focus from individual behavior change to the orchestration of care relationships.

### 4.1 Social interactions are a key driver of nutritional behavior

The social context of mealtimes is often overlooked in clinical approaches to malnutrition, yet it plays a crucial role in shaping older adults' nutritional behavior. Eating is not merely a physiological act of nutrient intake but also a deeply social practice embedded in relationships, routines, and cultural expectations. The concept of commensality, the practice of eating together, has long been recognized as a central mechanism for organizing and sustaining food practices. Shared meals provide structure to daily life, stimulate appetite, and reinforce a sense of belonging (58–60).

Research shows that eating alone is associated with reduced appetite, lower motivation to cook, and decreased dietary intake among older adults. Meals shared with others tend to last longer, involve greater food variety, and encourage higher food intake than solitary eating (59, 60). In addition, the presence of others can transform eating from a purely functional activity into a meaningful social occasion that reinforces identity, participation, and enjoyment. Conversely, when meals are experienced in isolation, older adults may lose the motivation to prepare meals, skip eating occasions, or reduce portion sizes (61, 62).

The dietitians in our study emphasized this social dimension of eating. Participants observed that when older adults eat alone, they often stop making an effort to cook or to create a pleasant mealtime setting. Consequently, dietitians frequently encourage members of the broader care network, such as family members, neighbors, or informal caregivers, to join older adults during meals whenever possible. Shared meals can increase enjoyment, stimulate appetite, and support adherence to dietary advice (59, 60).

However, facilitating such social interaction during mealtimes is not always straightforward. Informal caregivers may struggle to balance caregiving with work and family obligations. Similarly, home care professionals are typically unable to participate in mealtimes due to strict care regulations, task-oriented care structures, and time constraints. As a result, opportunities for shared meals are often limited.

Dietitians, therefore, sometimes recommend that older adults participate in communal dining initiatives, such as neighborhood meal programs or day-care facilities, which can provide both nutritional support and social engagement. While such initiatives can be effective, their availability is uneven, and access may be limited by mobility constraints, transportation barriers, or geographical disparities. Consequently, many older adults continue to experience meals in relative isolation.

These findings suggest that addressing malnutrition requires attention not only to nutritional intake but also to the social ecology of eating. Nutritional behavior is shaped by the relational and environmental conditions in which meals occur. When the social dimension of eating is absent, motivation to eat may decline even when adequate food and dietary advice are available.

Whether and to what extent SARs may play a positive role in the social aspects of eating depends on how they are positioned within the broader social ecology of mealtimes. Our findings suggest that dietitians do not primarily envision SARs as substitutes for human companionship, but rather as facilitators of meaningful eating experiences. Participants described opportunities for SARs to encourage shared meals, stimulate conversation, provide gentle social encouragement, and make solitary eating moments feel less routine and isolating. At the same time, they emphasized that the value of these interactions depends on preserving rather than replacing opportunities for genuine human connection.

Recent developments in SAR research increasingly emphasize companionship, anthropomorphic interaction, and affective engagement as key design goals, while comparatively less attention is paid to how these social qualities contribute to concrete care tasks and clinical outcomes (71, 72). This may point to a false dichotomy between instrumental and relational assistance. Our findings instead suggest that the social qualities of SARs derive much of their value precisely because they support functional goals. Conversation, encouragement, humour, and companionship are not merely pleasant additions to nutritional care; they can become mechanisms through which motivation, adherence, and the enjoyment of eating are sustained. In this sense, commensality should not be understood as an alternative to nutritional support, but as one of the pathways through which nutritional care becomes effective.

### 4.2 The potential of socially assistive robots as support in continuity of nutritional care

The findings highlight the complex context in which dietitians provide nutritional care. Consistent with previous research, fragmented collaboration, unclear role division, and organizational constraints continue to challenge the delivery of integrated nutritional care (10, 14, 22). At the same time, dietitians strive to translate dietary advice into meaningful and achievable actions while tailoring recommendations to older adults' individual needs, preferences, and changing circumstances. As also emphasized in previous research, such personalization is essential for supporting behaviour change and improving nutritional outcomes (57).

The findings indicate that dietitians see the value of SARs as supportive tools within the care process. Dietitians expect a SAR to reinforce dietary advice, provide motivational support, deliver information gradually, and facilitate communication and collaboration within the care network. Such support may help older adults to understand and adopt the dietary advice. SARs can support behavior change by using reminders, encouragement, continuous positive reflection, and offering long-term engagement, as improving nutritional intake requires perseverance and time.

While prior work positions SARs primarily as behavior-change tools (24, 32–34), our findings extend this view by showing that their perceived value lies equally in mediating social and collaborative processes within the care network. Their capacity for long-term engagement may be particularly valuable in helping older adults adhere to advice between consults by reinforcing it and providing motivational support (36, 37, 63, 64).

While dietitians primarily envisioned SARs as extending their support beyond consultations by reinforcing advice and encouraging healthy routines, participants also identified a more subtle role in supporting shared decision-making. Rather than participating in decisions themselves, SARs could help prepare and enrich conversations between the dietitian and the older adult by monitoring progress, prompting reflection on everyday experiences, and making relevant information available during consultations. In this way, SARs may help create the conditions for more meaningful shared decision-making, enabling older adults to participate more actively in discussions about their nutritional care while leaving interpretation, deliberation, and treatment decisions firmly with the dietitian. This aligns with literature emphasizing that shared decision-making depends not only on professional expertise but also on patients' ability to reflect on their experiences, express their preferences, and engage as active partners in care (65, 66).

### 4.3 Critical balance between a SAR and human-to-human contact

Whereas the previous section highlighted the potential of SARs as supportive technology within the nutritional care process, our findings also show a tension in how the technology's role is positioned. SARs are often proposed as social companions that may reduce loneliness during meal moments. While participants acknowledged this potential, it raises important ethical questions about the role of technology in socially meaningful contexts, such as shared meals. As was argued above, eating is not merely a functional activity but a social practice that carries emotional and relational significance. Replacing, or appearing to replace, human presence with a digital entity, therefore, raises concerns about the authenticity of social interaction and the risk that technological companionship may substitute for human contact rather than complement it.

According to participants, a primary function of a SAR should include mealtime reminders, a feature common in many behavior-change technologies. However, reminders alone are unlikely to produce meaningful changes in nutritional behavior. Sustained behavior change requires a combination of intrinsic and extrinsic motivation, knowledge, perceived benefits, and the practical ability to implement changes in everyday routines (68, 69). In this sense, reminders may support adherence but cannot replace the relational and motivational work that dietitians perform during consultations.

The dietitians in our study repeatedly emphasized that older adults value being listened to and understood as individuals. From their perspective, a SAR would be acceptable only if it could support meaningful interaction rather than merely deliver scripted prompts. This expectation introduces another ethical tension. Designing SARs that appear attentive, empathetic, or emotionally responsive may improve engagement, but it may also create ambiguity about the nature of the relationship between the user and the technology. Older adults might attribute social understanding or emotional responsiveness to a system that, in reality, relies on programmed responses or algorithmic pattern recognition. Such dynamics raise questions about transparency, emotional authenticity, and the potential for users to develop misplaced expectations about what the technology can actually understand or provide (61).

Participants also pointed to the risk that SARs might blur boundaries between technological support and professional care. While SARs may provide reminders, encouragement, or daily structure, there is a concern that reliance on these systems could gradually shift responsibilities away from human caregivers. In particular, overreliance on automated encouragement or monitoring could unintentionally undermine older adults' sense of self-efficacy or autonomy if the system becomes overly directive. Thus, negatively influencing their active role in managing their dietary behavior. From an ethical perspective, this underscores the importance of ensuring that SARs support rather than replace older adults' agency in managing their own nutritional behavior. This is also discussed by Grote and Berens, AI-supported systems such as SARs can negatively affect the older adults' sense of autonomy (67).

At the same time, as highlighted in the previous section, the dietitians clearly recognized the potential benefits of SARs as supportive tools within the care network. They expressed openness toward systems that could reinforce advice, provide reminders, share information, and reduce the threshold for communication between consultations. These findings suggest that SARs may be positioned as extensions of care that help sustain engagement and awareness between professional encounters. Crucially, however, participants stressed that defining, adapting, and interpreting dietary advice must remain the dietitian's responsibility. Professional judgement, built on personal interaction and contextual understanding, was considered irreplaceable in determining when and how interventions should occur.

Interestingly, the discussions also revealed a tension in how dietitians envision the role of such systems. On the one hand, participants insisted that SARs should not interfere with the clinical decision-making process. On the other hand, they suggested that SARs could alert them when additional attention to a client might be needed. Generating such alerts would require some interpretive capability, for example, detecting patterns in nutritional intake, mood, or behavioural changes. This illustrates a broader ethical and practical challenge: technologies designed to remain supportive and non-directive must nonetheless perform some level of interpretation in order to be useful. These findings resonate with the work of Lukkien et al. (70), demonstrating that AI-supported systems can support healthcare professionals in early signaling and emerging needs by, for example, monitoring behavior patterns. Importantly, in their work, they emphasize that these systems should serve as decision-support tools, not decision-makers.

Although this study focused exclusively on dietitians supporting community-dwelling older adults, the challenges addressed, such as malnutrition, cognitive decline, motivation, and the social nature of eating, are also relevant to residential long-term care. However, the findings cannot be assumed to transfer directly to these settings, as meals, caregiving responsibilities, and interdisciplinary collaboration are organized differently.

These organizational differences may change the role that SARs could play. In community care, participants primarily envisioned SARs as bridging discontinuities between dietetic consultations and supporting older adults when professional or informal caregivers are absent. In residential long-term care, professional caregivers are more continuously present, meals may be embedded in organized routines, and nutritional care is distributed across an interdisciplinary team. The composition and involvement of the care network may also differ, with professional caregivers taking a more continuous role in everyday nutritional care and informal caregivers occupying a different position than in community settings. Consequently, the potential value of SARs may lie less in compensating for the absence of care and more in complementing existing care routines, for example by reinforcing individualized nutritional goals, supporting resident engagement during meals, or facilitating shared awareness of nutritional intake and behavioral changes across professionals.

The social role of SARs may also differ. Although communal eating opportunities may be more readily available in residential settings, eating in the presence of others does not necessarily result in meaningful social interaction, enjoyment, or adequate intake, particularly for residents experiencing cognitive or physical decline. SARs might therefore support engagement within existing mealtime settings rather than functioning primarily as companions during solitary meals.

At the same time, introducing SARs into an environment with continuous professional caregiving raises additional questions about role boundaries, responsibility, workflow integration, and the risk that technology displaces rather than supports human interaction. Future research should therefore examine whether and how the roles identified in this community-based study translate to residential long-term care, involving residents, dietitians, nursing staff, other care professionals, and informal caregivers in evaluating how SARs could complement existing practices without disrupting valued human relationships.

In conclusion, these findings highlight the delicate balance required when integrating socially assistive technologies into nutritional care practices. On the one hand, technological support is increasingly necessary in a context of limited professional time and growing care demands. On the other hand, participants were strongly concerned that technology should not erode the relational aspects of care that are central to effective nutritional support. Rather than replacing human contact, SARs should therefore be positioned as tools that help sustain relationships, support communication, and reinforce professional care without displacing the human actors who ultimately remain responsible for care decisions.

## 5 Conclusion

This study explored how dietitians conceptualize the role of socially assistive robots within malnutrition care using a research-through-design approach. Unlike much prior work focusing on individual user–robot interaction, our findings highlight that SARs should be designed to operate within a multi-actor care network, mediating communication between dietitians, older adults, and informal caregivers. In other words, rather than positioning SARs as standalone interventions, our findings highlight their potential as supportive extensions of care that reinforce advice, facilitate communication, and sustain engagement between consultations.

Crucially, the integration of SARs is shaped by persistent tensions between technological support and human care, particularly regarding autonomy, responsibility, and the social nature of eating. These findings suggest that the value of SARs lies not in replacing care but in carefully augmenting the relational and collaborative dynamics within the care network.

Future work should therefore move beyond functionality-driven design and investigate how SARs can be responsibly embedded in real-world care practices across different care settings, while preserving the human aspects that remain central to effective nutritional support. Rather than treating functional and social support as separate design objectives, future research should explore how relational qualities such as encouragement, companionship, and shared engagement can themselves become mechanisms for supporting nutritional behaviour, adherence, and collaborative care.

### 5.1 Design implications for SARs in malnutrition care

Based on our findings, we derive several design implications for socially assistive robots in malnutrition care.

First, SARs should be designed as extensions of professional care rather than autonomous agents. Dietitians emphasized that interpreting and adapting dietary advice requires contextual understanding and professional judgement. SARs should therefore reinforce and operationalize advice, while leaving decision-making authority with the dietitian.

Second, SARs should support the translation of dietary advice into everyday behavior. Rather than providing generic recommendations, SARs should deliver context-specific prompts that align with daily routines, helping older adults to implement advice in manageable steps.

Third, SARs should be designed to mediate collaboration within the care network. Our findings highlight that malnutrition care is inherently distributed across multiple actors. SARs may therefore function as coordination tools that support communication, signal emerging issues, and facilitate shared awareness between dietitians, older adults, and informal caregivers.

Fourth, SAR interactions should support autonomy rather than impose control. Motivational prompts should remain encouraging and non-directive, avoiding overreliance or reduced self-efficacy.

Finally, transparency in the role and functioning of SARs is essential. Designers should clearly distinguish between dietitian-originated advice and system-generated prompts to prevent confusion and maintain trust.

Together, these implications suggest that designing SARs for malnutrition care requires moving beyond functionality to carefully consider their role within relational and collaborative care processes.

## 6 Future work & limitations

This study explored the role of SARs within nutritional care; future research is needed to detail the functionality of SARs as they are explored and evaluated in real-life settings, while considering ethical tensions regarding overreliance, autonomy, commensality, and blurring boundaries. Given the importance of shared meal moments and the social nature of eating together, future studies should specifically examine how SARs are positioned to support human interaction. Whereas current research focused on the dietitian's perspective, the stakeholders within the care network need to be further involved to explore the role and positioning of SARs within the broader care network. These new perspectives will help refine the role and expectations towards SARs. With these different perspectives, we aim to gain insights not only into how each separate actor can be supported but also into how to improve shared awareness and collaboration among care network members (see Figure 8).

**Figure 8:**
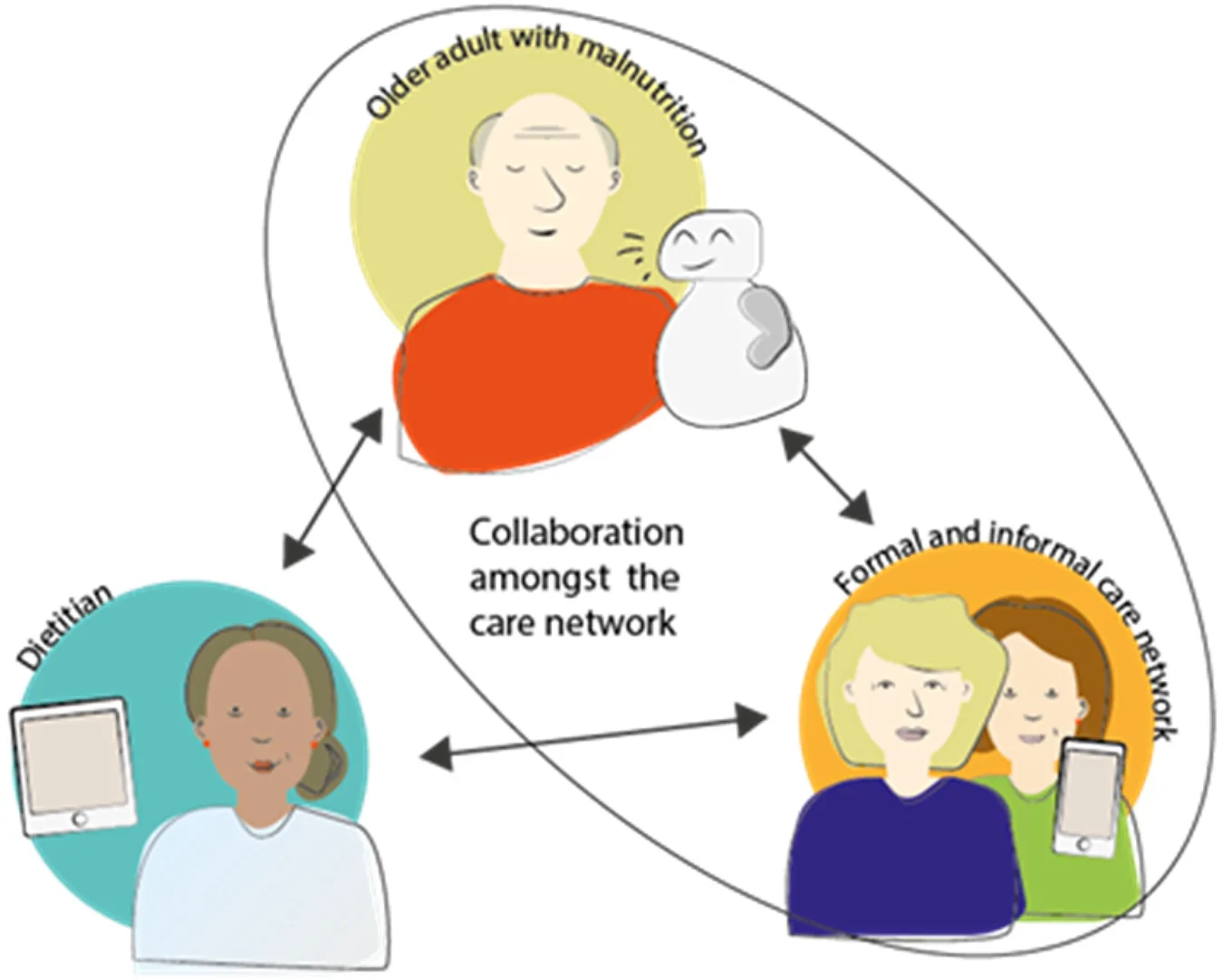
Future focus will be on older adults and their care network needs, as well as the collaboration amongst the care network members.

This study has several limitations. First, the sample size was relatively small, and participants were recruited through professional networks, which may have introduced recruitment bias. Dietitians with an interest in innovation may have been more likely to participate, potentially underrepresenting more skeptical perspectives towards technology. In addition, the RtD sessions were conducted in dyads with dietitians from the same practice. Although this format stimulated discussion and reflection, participants may have influenced each other's perspectives during the generative design activities. In some dyads, one participant held a more senior or supervisory role within the practice, which may have influenced the extent to which colleagues felt comfortable expressing differing opinions or critical experiences. Throughout the session, participants completed the probe activities individually while engaging in discussion, allowing each participant to express their own ideas rather than working towards a shared response. In addition, the facilitator actively encouraged both participants to contribute equally and share their own perspectives. Nevertheless, these interpersonal dynamics may have shaped the discussions and should be considered when interpreting the findings.

Second, the study did not include the perspectives of older adults or other members of the care network, which limits the generalizability of the findings. Third, the RtD approach is inherently exploratory and speculative in nature. Therefore, the findings should be interpreted as generative insights that help explore possible roles and tensions of SARs, rather than as predictions of real-world implementation. Finally, all participating dietitians provided nutritional care to community-dwelling older adults. The findings therefore reflect the organization and challenges of community-based nutritional care and should not be generalized to residential long-term care, where caregiving, mealtimes, and interdisciplinary collaboration are organized differently.

## 7 Commercial interests

Martijn Vastenburg is the founder of ConnectedCare, a company developing the SAR Lizz. The publication has not been restricted in any way in view of commercial interests.

## Data Availability

The datasets presented in this article are not readily available because the datasets generated and analyzed during the current study are not publicly available due to privacy and confidentiality restrictions, as they contain identifiable qualitative data. However, anonymized data may be made available by the corresponding author upon reasonable request and in accordance with ethical approval. The metadata are accessible via the 4TU.ResearchData repository. Requests to access the datasets should be directed to j.alberts@tue.nl.

## References

[1] Leij-Halfwerk S Verwijs MH Van Houdt S Borkent JW Guaitoli PR Pelgrim T. Prevalence of protein-energy malnutrition risk in European older adults in community, residential and hospital settings, according to 22 malnutrition screening tools validated for use in adults ≥65 years: a systematic review and meta-analysis. Maturitas. (2019) 126:80–9. 10.1016/j.maturitas.2019.05.006 31239123

[2] Volkert D Kiesswetter E Cederholm T Donini LM Eglseer D Norman K. Development of a model on determinants of malnutrition in aged persons: a MaNuEL project. Gerontol Geriatr Med. (2019) 5:2333721419858438. 10.1177/2333721419858438 31259204 PMC6589946

[3] Leslie W Hankey C. Aging, nutritional status and health. Healthcare. (2015) 3(3):648–58. 10.3390/healthcare3030648 27417787 PMC4939559

[4] Cederholm T Jensen GL Correia MITD Gonzalez MC Fukushima R Higashiguchi T. GLIM criteria for the diagnosis of malnutrition: a consensus report from the global clinical nutrition community. Clin Nutr. (2019) 38(1):1–9. 10.1016/j.clnu.2018.08.002 30181091

[5] Evans C. Malnutrition in the elderly: a multifactorial failure to thrive. Perm J. (2005) 9(3):38–41. 10.7812/TPP/05-056 22811627 PMC3396084

[6] Volkert D Beck AM Cederholm T Cereda E Cruz-Jentoft A Goisser S. Management of malnutrition in older patients: current approaches, evidence and open questions. J Clin Med. (2019) 8(7):974. 10.3390/jcm8070974 31277488 PMC6678789

[7] Rasheed S Woods RT. Malnutrition and quality of life in older people: a systematic review and meta-analysis. Ageing Res Rev. (2013) 12(2):561–6. 10.1016/j.arr.2012.11.003 23228882

[8] Marshall S Bauer J Isenring E. The consequences of malnutrition following discharge from rehabilitation to the community: a systematic review of current evidence in older adults. J Hum Nutr Diet. (2014) 27(2):133–41. 10.1111/jhn.12167 24289811

[9] Whitelock E Ensaff H. On your own: older adults’ food choice and dietary habits. Nutrients. (2018) 10(4):413. 10.3390/nu10040413 29584644 PMC5946198

[10] Ziylan C Haveman-Nies A Van Dongen EJI Kremer S De Groot LCPGM. Dutch nutrition and care professionals’ experiences with undernutrition awareness, monitoring, and treatment among community-dwelling older adults: a qualitative study. BMC Nutr. (2015) 1:38. 10.1186/s40795-015-0034-6

[11] Beelen J Vasse E Ziylan C Janssen N De Roos NM De Groot LCPGM. Undernutrition: who cares? Perspectives of dietitians and older adults on undernutrition. BMC Nutr. (2017) 3:24. 10.1186/s40795-017-0144-4 32153806 PMC7050937

[12] Lacey K Pritchett E. Nutrition care process and model: ADA adopts road map to quality care and outcomes management. J Am Diet Assoc. (2003) 103(8):1061–72. 10.1016/S0002-8223(03)00971-4 12891159

[13] Nieuwenhuizen WF Weenen H Rigby P Hetherington MM. Older adults and patients in need of nutritional support: review of current treatment options and factors influencing nutritional intake. Clin Nutr. (2010) 29(2):160–9. 10.1016/j.clnu.2009.09.003 19828215

[14] Verwijs MH Puijk-Hekman S Van Der Heijden E Vasse E De Groot LCPGM De Van Der Schueren MAE. Interdisciplinary communication and collaboration as key to improved nutritional care of malnourished older adults across health-care settings: a qualitative study. Health Expect. (2020) 23(5):1096–107. 10.1111/hex.13075 32525265 PMC7696200

[15] Fleurke M Voskuil DW Beneken Genaamd Kolmer DM. The role of the dietitian in the management of malnutrition in the elderly: a systematic review of current practices. Nutr Diet. (2020) 77(1):60–75. 10.1111/1747-0080.12546 31157519

[16] Ten Cate D Mellema M Ettema RGA Schuurmans MJ Schoonhoven L. Older adults’ and their informal caregivers’ experiences and needs regarding nutritional care provided in the periods before, during and after hospitalization: a qualitative study. J Nutr Gerontol Geriatr. (2021) 40(2–3):80–107. 10.1080/21551197.2021.1906822 33835889

[17] Minkman MM Zonneveld N Hulsebos K Van Der Spoel M Ettema R. The renewed development model for integrated care: a systematic review and model update. BMC Health Serv Res. (2025) 25(1):434. 10.1186/s12913-025-12610-2 40140980 PMC11938726

[18] Doornebosch AJ Smaling HJA Achterberg WP. Interprofessional collaboration in long-term care and rehabilitation: a systematic review. J Am Med Dir Assoc. (2022) 23(5):764–77.e2. 10.1016/j.jamda.2021.12.028 35065048

[19] Fitzpatrick G Ellingsen G. A review of 25 years of CSCW research in healthcare: contributions, challenges and future agendas. Comput Support Coop Work. (2013) 22(4–6):609–65. 10.1007/s10606-012-9168-0

[20] Rechel B Grundy E Robine J-M Cylus J Mackenbach JP Knai C. Ageing in the European union. Lancet. (2013) 381(9874):1312–22. 10.1016/S0140-6736(12)62087-X 23541057

[21] Lipszyc B Sail E Xavier A Long-term care: need, use and expenditure in the EU-27. European commission directorate-general for economic and financial affairs. Economic papers No. 469 (2012).

[22] Dent E Hoogendijk EO Wright ORL. New insights into the anorexia of ageing: from prevention to treatment. Curr Opin Clin Nutr Metab Care. (2019) 22(1):44–51. 10.1097/MCO.0000000000000525 30394894

[23] Marx W Kelly JT Crichton M Craven D Collins J Mackay H. Is telehealth effective in managing malnutrition in community-dwelling older adults? A systematic review and meta-analysis. Maturitas. (2018) 111:31–46. 10.1016/j.maturitas.2018.02.012 29673830

[24] Łukasik S Tobis S Wieczorowska-Tobis K Suwalska A. Could robots help older people with age-related nutritional problems? Opinions of potential users. Int J Environ Res Public Health. (2018) 15(11):2535. 10.3390/ijerph15112535 PMC6266220 30424561

[25] Bonilla C Brauer P Royall D Keller H Hanning RM DiCenso A. Use of electronic dietary assessment tools in primary care: an interdisciplinary perspective. BMC Med Inform Decis Mak. (2015) 15:14. 10.1186/s12911-015-0138-6 25886381 PMC4364652

[26] Borkent JW Naumann E Vasse E Van Der Heijden E De Van Der Schueren MAE. Prevalence and determinants of undernutrition in a sample of Dutch community-dwelling older adults: results from two online screening tools. Int J Environ Res Public Health. (2019) 16(9):1562. 10.3390/ijerph16091562 31060212 PMC6539798

[27] Lucassen DA Lasschuijt MP Camps G Van Loo EJ Fischer ARH De Vries RAJ. Short and long-term innovations on dietary behavior assessment and coaching: present efforts and vision of the pride and prejudice consortium. Int J Environ Res Public Health. (2021) 18(15):7877. 10.3390/ijerph18157877 34360170 PMC8345591

[28] Tufano M Lasschuijt M Chauhan A Feskens EJM Camps G. Capturing eating behavior from video analysis: a systematic review. Nutrients. (2022) 14(22):4847. 10.3390/nu14224847 36432533 PMC9697383

[29] Lasschuijt MP Mars M Stieger M Miquel-Kergoat S De Graaf C Smeets PAM. Comparison of oro-sensory exposure duration and intensity manipulations on satiation. Physiol Behav. (2017) 176:76–83. 10.1016/j.physbeh.2017.02.003 28174138

[30] Cohen R Fernie G Roshan Fekr A. Fluid intake monitoring systems for the elderly: a review of the literature. Nutrients. (2021) 13(6):2092. 10.3390/nu13062092 34205234 PMC8233832

[31] Soufargi S Niewiadomski R Fong H Mancini M. A social robot companion for individuals eating alone. In: Companion Proceedings of the 30th International Conference on Intelligent User Interfaces (2025), 5–8 10.1145/3708557.3716340

[32] Niewiadomski R Bruijnes M Huisman G Gallagher CP Mancini M. Social robots as eating companions. Front Comput Sci. (2022) 4:909844. 10.3389/fcomp.2022.909844

[33] Fujii A Okada K Inaba M. A study on design and moving behavior of meal partner robots that can perform eating behavior expression. Adv Robot. (2024) 38(7):1441–56. 10.1080/01691864.2024.2388115

[34] McColl D Nejat G. Meal-time with a socially assistive robot and older adults at a long-term care facility. J Hum Robot Interact. (2013) 2(1):152–71. 10.5898/JHRI.2.1.McColl

[35] Bouwhuis DG. Current use and possibilities of robots in care. Gerontechnology. (2016) 15(4):198–208. 10.4017/gt.2016.15.4.003.00

[36] Ciuffreda I Amabili G Casaccia S Benadduci M Margaritini A Maranesi E. Design and development of a technological platform based on a sensorized social robot for supporting older adults and caregivers: gUARDIAN ecosystem. Int J Soc Robot. (2025) 17(5):803–22. 10.1007/s12369-023-01038-5

[37] Casaccia S Revel GM Scalise L Bevilacqua R Rossi L Paauwe RA. Social robot and sensor network in support of activity of daily living for people with dementia. In: Dementia Lab Conference (Cham: Springer International Publishing) (2019), 1117, 128–35 10.1007/978-3-030-33540-3_12

[38] Boumans R Van Meulen F Hindriks K Neerincx M Olde Rikkert M. A feasibility study of a social robot collecting patient-reported outcome measurements from older adults. Int J Soc Robot. (2020) 12(1):259–66. 10.1007/s12369-019-00561-8

[39] Weber P Krings K Nießner J Brodesser S Ludwig T. FoodChattAR: exploring the design space of edible virtual agents for human-food interaction. In: Proceedings of the 2021 ACM Designing Interactive Systems Conference (2021), 638–50. 10.1145/3461778.3461998

[40] Nunes F Verdezoto N Fitzpatrick G Kyng M Grönvall E Storni C. Self-care technologies in HCI: trends, tensions, and opportunities. ACM Trans Comput-Hum Interact. (2015) 22(6):1–45. 10.1145/2803173

[41] Green M Dao D Moyle W. Design attributes of socially assistive robots for people with dementia: a systematic review. Int J Soc Robot. (2024) 16(2):415–27. 10.1007/s12369-023-01087-w

[42] Fischer B Peine A Östlund B. The importance of user involvement: a systematic review of involving older users in technology design. Gerontologist. (2020) 60(7):e513–23. 10.1093/geront/gnz163 31773145 PMC7491439

[43] De Carolis B Macchiarulo N D'Errico F Palestra G. Social robots in cognitive interventions: advances, problems and perspectives. In: Proceedings of the 13th Biannual Conference of the Italian SIGCHI Chapter (2021), 376–80. 10.1145/3461615.3485421

[44] Stappers PJ. Doing design as a part of doing research. In: Michel R, editor. Design Research now. Basel: Birkhäuser Basel (2007). p. 81–91. 10.1007/978-3-7643-8472-2_6

[45] Sanders EBN Stappers PJ. Convivial Toolbox: Generative Research for the Front End of Design. Amsterdam: BIS Publishers (2012). p. 272. ISBN: 978-90-6369-284-1.

[46] Visser FS Stappers PJ Van Der Lugt R Sanders EBN. Contextmapping: experiences from practice. CoDesign. (2005) 1(2):119–49. 10.1080/15710880500135987

[47] Rosson MB Gilmore D Zimmerman J Forlizzi J Evenson S. Research through design as a method for interaction design research in HCI. In: Proceedings of the SIGCHI Conference on Human Factors in Computing Systems (New York, NY: Association for Computing Machinery) (2007), 493–502. 10.1145/1240624.1240704

[48] Zimmerman J Forlizzi J Evenson S. Research through design as a method for interaction design research in HCI. In: Proceedings of the SIGCHI Conference on Human Factors in Computing Systems (2007), 493–502

[49] Alberts JW Vastenburg MH Nap HH IJsselsteijn WA. Towards healthy diets and sustainable nutritional behavior: identifying design opportunities for technology-supported malnutrition care. J Ageing Longev. (2026) 6(3):50. 10.3390/jal6030050

[50] PARO Robots. PARO Therapeutic Robot. Available online at: https://www.parorobots.com/ (Accessed July 16, 2026).

[51] Tinybots. Ontmoet Tessa: jouw persoonlijke zorgassistent. Available online at: https://www.tinybots.nl/ (Accessed July 16, 2026).

[52] SARA Robotics. SARA Robotics. Available online at: https://zorgenmetsara.nl/ (Accessed July 16, 2026).

[53] Braun V Clarke V. Reflecting on reflexive thematic analysis. Qual Res Sport Exerc Health. (2019) 11(4):589–97. 10.1080/2159676X.2019.1628806

[54] Byrne D. A worked example of Braun and Clarke’s approach to reflexive thematic analysis. Qual Quant. (2022) 56:1391–412. 10.1007/s11135-021-01182-y

[55] VERBI Software. MAXQDA. Version 26 [Computer Software]. Berlin: VERBI Software (2026). Available online at: https://www.maxqda.com.

[56] Graafmans JAM Brouwers T. GerontechnologyTM, the modelling of normal aging. In: Proceedings of the Human Factors Society Annual Meeting (Los Angeles, CA: SAGE Publications) (1989), 33, 3, 187–9. 10.1177/15419312890330030

[57] Rusu A Randriambelonoro M Perrin C Valk C Álvarez B Schwarze AK. Aspects influencing food intake and approaches towards personalising nutrition in the elderly. J Popul Ageing. (2020) 13(2):239–56. 10.1007/s12062-019-09259-1

[58] Björnwall A Mattsson Sydner Y Koochek A Neuman N. Eating alone or together among community-living older people: a scoping review. Int J Environ Res Public Health. (2021) 18(7):3495. 10.3390/ijerph18073495 PMC8036467 33801775

[59] Herman CP. The social facilitation of eating: a review. Appetite. (2015) 86:61–73. 10.1016/j.appet.2014.09.016 25265153

[60] Vesnaver E Keller HH. Social influences and eating behavior in later life: a review. J Nutr Gerontol Geriatr. (2011) 30(1):2–23. 10.1080/01639366.2011.545038 23286638

[61] Wang F Perugia G Feng Y IJsselsteijn W Social robots for people with dementia: a literature review on deception from design to perception. *arXiv* [Preprint]. *arXiv:2507.00963*. (2025).

[62] Locher JL Burgio KL Yoels WC Ritchie S. The social significance of food and eating in the lives of older recipients of meals on wheels. J Nutr Elder. (1998) 17(2):15–33. 10.1300/J052v17n02_02

[63] Hofstede BM Askari SI Van Hoesel TRC Cuijpers RH De Witte LP IJsselsteijn WA. Huggable integrated socially assistive robots: exploring the potential and challenges for sustainable use in long-term care contexts. Front Robot AI. (2025) 12:1646353. 10.3389/frobt.2025.1646353 41170301 PMC12569430

[64] Søraa RA Tøndel G Kharas MW Serrano JA. What do older adults want from social robots? A qualitative research approach to human-robot interaction studies. Int J Soc Robot. (2023) 15(3):411–24. 10.1007/s12369-022-00914-w

[65] Pel-Littel RE Snaterse M Teppich NM Buurman BM Van Etten-Jamaludin FS Van Weert JCM. Barriers and facilitators for shared decision making in older patients with multiple chronic conditions: a systematic review. BMC Geriatr. (2021) 21(1):112. 10.1186/s12877-021-02050-y 33549059 PMC7866443

[66] Dyrstad DN Testad I Aase K Storm M. A review of the literature on patient participation in transitions of the elderly. Cogn Technol Work. (2015) 17(1):15–34. 10.1007/s10111-014-0300-4

[67] Grote T Berens P. On the ethics of algorithmic decision-making in healthcare. J Med Ethics. (2020) 46(3):205–11. 10.1136/medethics-2019-105586 31748206 PMC7042960

[68] Michie S van Stralen MM West R. The behaviour change wheel: a new method for characterising and designing behaviour change interventions. Implement Sci. (2011) 6:42. 10.1186/1748-5908-6-42. 21513547 PMC3096582

[69] Maibaum A Bischof A Hergesell J Lipp B. A critique of robotics in health care. AI Soc. (2022) 37:467–77. 10.1007/s00146-021-01206-z

[70] Lukkien DRM. Responsible artificial intelligence in long-term care: from concept to context [doctoral dissertation]. Utrecht: Utrecht University (2025). 10.33540/3201

[71] Ahmed E Buruk OO Hamari J. (2024). Human–robot companionship: current trends and future agenda. Int J Soc Robot. 16(8):1809-60.

[72] Cameron CD Wagner AR Orlandi M Hadjiandreou E Oates IG Anderson S. (2025). Empathy for and From Embodied Robots: An Interdisciplinary Review. Curr Dir Psychol Sci. (2025):09637214251392861.

